# Simple Synthesis of Hydroxyl and Ethylene Functionalized Aromatic Polyamides as Sizing Agents to Improve Adhesion Properties of Aramid Fiber/Vinyl Epoxy Composites

**DOI:** 10.3390/polym9040143

**Published:** 2017-04-20

**Authors:** Minglin Qin, Haijuan Kong, Kang Zhang, Cuiqing Teng, Muhuo Yu, Yaozu Liao

**Affiliations:** 1 State Key Laboratory for Modification of Chemical Fibers and Polymer Materials & College of Materials Science and Engineering, Donghua University, Shanghai 201620, China; 1139029@mail.dhu.edu.cn (M.Q.); xingyuhuap6suc@163.com (K.Z.); 2 School of Materials Engineer, Shanghai University of Engineering Science, Shanghai 201620, China; konghaijuan@sues.edu.cn

**Keywords:** aramid fibers, sizing agents, vinyl epoxy composites, adhesion properties

## Abstract

To improve interfacial adhesion between aramid fibers and vinyl epoxy resins, a series of hydroxyl and ethylene-functional aromatic polyamides ((ClPPTA)_m_-R′) with different chain segments were successfully synthesized via a one-pot low-temperature polycondensation. The hydroxyl and ethylene-functional aromatic polyamides were characterized by Fourier transform infrared spectroscopy (FT-IR), solid-state ^13^C CP/MAS nuclear magnetic resonance spectroscopy (^13^C CP/MAS NMR), thermal gravimetric analysis (TGA), and wide-angle X-ray diffraction (WXRD). The contact angle of the hydroxyl and ethylene-functional aromatic polyamides films were measured. The hydroxyl and ethylene-functional aromatic polyamides were used as the sizing agents for aramid fiber/vinyl epoxy composites. The surface chemical composition and morphology of the unsized and sized fibers were identified using X-ray photoelectron spectroscopy (XPS) and scanning electron microscopy (SEM). The interfacial adhesion between aramid fibers and vinyl epoxy composites was investigated by the micro-debond tests. The results showed that the interfacial shear strength between the sized aramid fibers and vinyl epoxy composites was greatly improved.

## 1. Introduction

The wholly aromatic polyamide fibers have been applied for many promising industrial applications owing to their high melting point, high glass-transition temperature, high heat resistance, flame retardance, and good chemical shielding [1]. Poly(*p*-phenylene terphthalamide) (PPTA) is one of the most studied aramid polyamides [2]. The PPTA exhibits excellent thermal stability, flame resistance, low density, high strength and modulus because of its rod-like chemical structures, and strong intermolecular interactions [3,4]. The PPTA can be processed in the form of fibers such as Kevlar fibers, which have been used as reinforcing compositions for composite materials and applied in many fields such as aeronautics and astronautics, military, automobiles, and sports [2,3,4,5,6]. To realize the purposes mentioned above, however, one of the biggest challenges is that the Kevlar fibers exhibit a low affinity for interfacial adhesion towards the resin matrix. In order to overcome this difficulty, surface modifications of aramid fibers, in order to improve the adhesion, have been done, including plasma treatment [7,8,9,10], chemical etching [11], chemical grafting [12], oxidation treatment [13], coating [14], and physical irradiation [15]. These methods have achieved some successes in improving the adhesion, but most of them lead to a partial loss of the tensile strength of the fibers. The introduction of a sizing agent is reported as an effective way to improve the interfacial adhesion of epoxy/rubber composite materials [14,16,17,18,19,20,21,22]. It is also proposed as an effective approach to improve the compatibility between aramid fiber and other polymers via introducing various functional groups into an aromatic polyamide [23,24,25,26,27,28,29]. Wan and co-workers introduced allyl and hydroxyl groups into the aramid fiber surface [21,22], remarkably enhancing the interfacial adhesion between PPTA fibers and epoxy/rubber composites. Unfortunately, there are many limitations of this method such as harsh reaction conditions and multi-step synthesis processes needed. 

**Scheme 1:**
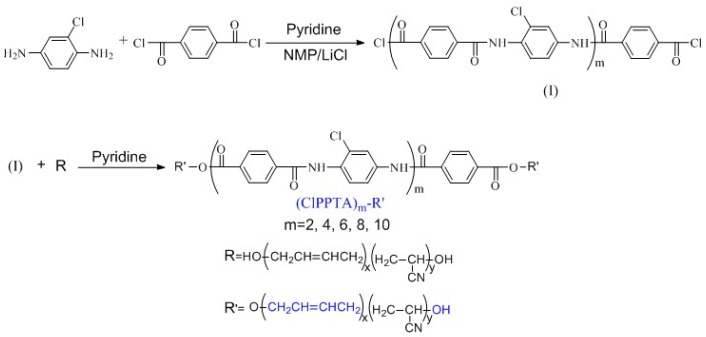
Synthetic route of the hydroxyl and ethylene-functional aromatic polyamides i.e., ((ClPPTA)_m_-R′).

In this paper, we reported a simple one-pot method to synthesize a series of hydroxyl and ethylene-functional aromatic polyamides ((ClPPTA)_m_-R′) via a low-temperature polycondensation reaction by using 2-chloro-1,4-diaminobenzene, terphthaloyl chloride, and hydroxyl-terminated liquid nitrile rubber (HTBN) as precursors in polar organic solvents (Scheme 1). PPTA is difficult to dissolve in any organic solvents, chemical modification of PPTA is a plausible method to improve the polymer solubility. It has been suggested that the introduction of halogen groups onto the aromatic polyamide is an effective method to improve the solubility of the polymer [30]. HTBN not only can be used as adhesives for rubber, polyester and metal, but also as a toughening agent for the modification of epoxy resin [31]. Based on this view, HTBN was firstly introduced into the aromatic polyamide. The ((ClPPTA)_m_-R′) not only inherits the characteristics of HTBN, which can be used as the sizing agents to improve the interfacial adhesion of the vinyl epoxy resin composites, but also maintains excellent performance of aromatic polyamide that can strongly interact with aramid fibers by intermolecular hydrogen bonding. The structures of ((ClPPTA)_m_-R′) products were characterized by Fourier transform infrared spectroscopy (FT-IR), solid-state ^13^C CP/MAS nuclear magnetic resonance spectroscopy (^13^C CP/MAS NMR), X-ray diffraction (XRD), and thermal gravimetric analysis (TGA). The solubility and contact angle of the ((ClPPTA)_m_-R′) were tested and discussed. The surface chemical composition and morphology of original and modified fibers were estimated by X-ray photoelectron spectroscopy (XPS) and scanning electron microscopy (SEM). The interfacial shear strength (IFSS) of the aramid fibers and vinyl epoxy composites was investigated by the micro-debond tests, the results showed that the IFSS was greatly improved.

## 2. Materials and Methods

### 2.1. Materials

Terphthaloyl chloride (TPC, >99.9%) and *N*-methyl-2-pyrrolidone (NMP, >99.9%) were purchased from Hebei Silicon Valley Chemical Co., Ltd., Handan, China. 4 Å molecular sieve, lithium chloride anhydrous (>96%), H_2_SO_4_ (98%) and pyridine (>99.5%) were purchased from Sinopharm Chemical Reagent Co., Ltd., Shanghai, China. Hydroxyl-terminated liquid nitrile rubber (*M*_w_ = 2500–4000) was bought from Zibo Qilong Chemical Industry Co., Ltd., Zibo, China. Acetone was purchased from shanghai Ling Feng Chemical Reagent Co., Ltd., Shanghai, China. 2-Chloro-1,4-diaminobenzene(Cl-PPD, >99.9%) was purchased from Changzhou sunlight Pharmaceutical Co., Ltd., Changzhou, China. LiCl and 4 Å molecular sieve were dried at 400 °C in an oven for 8 h before use. NMP and pyridine were dried by stirring with CaH_2_ and distilled out under reduced pressure, then added in a 4 Å molecular sieve in order to remove trace water. The water content in NMP after this treatment is below 30 ppm, measured using a moisture-testing instrument. The aramid fibers (K49) with a diameter of 15 μm were supplied DuPont Co., Ltd., Senatobia, MS, USA. The fibers were extracted with acetone overnight and dried in a vacuum over at 60 °C for 6 h before use. Vinyl epoxy resin (MFE-2) was purchased from Sino Polymer Co., Ltd., Shanghai, China. Cobalt (II) naphthenate was bought from Sinopharm Chemical Reagent Co., Ltd. 2-Butanone peroxide was purchased from Shanghai Aladdin Biological Co., Ltd., Shanghai, China. The vinyl epoxy resin and curing agent at the ratio of 100:1.5:1 (Resin: 2-Butanone: Cobalt (II) naphthenate). All other reagents and solvents were used as received unless otherwise specified.

### 2.2. General Polymerization Procedure

Polycondensation experiments were performed according to the following procedures, Cl-PPD (4.97 g, 0.035 mol) and 129 mL of NMP containing 2.2 g LiCl were put in a flame-dried 250 mL three-necked round-bottom flask, with two necks equipped with a mechanical stir and a nitrogen flow, respectively. After Cl-PPD and LiCl were dissolved completely, the mixture was cooled in an ice bath at 0 °C for 10 min Afterward, TPC (9.14 g, 0.045 mol) was added and the reaction mixture was stirred at 0 °C for 30 min to obtain a black viscous solution containing acyl chloride-terminated poly(2-chlorine-1,4-phenylene terephthalamide) oligomer ((ClPPTA)_m_). Then the excess hydroxyl-terminated liquid nitrile rubber was slowly added, and the mixture was stirred at 70 °C for 3 h. After cooling to room temperature naturally, the suspension was poured into a large amount of deionized water, the precipitate was collected by cyclic filtration, as well as water and acetone washing for several times, until the excess hydroxyl-terminated liquid nitrile rubber was completely washed away. Finally, the products hydroxyl and ethylene-functional aromatic polyamides i.e., ((ClPPTA)_m_-R′) were dried under vacuum at 60 °C overnight. According to Carothers’ equation by simply varying the monomer ratio, every desired composition can be made [32]. The synthesis route of the ((ClPPTA)_m_-R′) is demonstrated in Scheme 1.

### 2.3. Films Fabrication

The PPTA and ((ClPPTA)_m_-R′) films were prepared according to the following procedures: (1) The PPTA polymer solution was prepared by dissolving the PPTA polymer in concentrated H_2_SO_4_ at 3 wt % solid content, and the ((ClPPTA)_m_-R′) was dissolved in NMP/LiCl at 1.5 wt % solid content; (2) the PPTA solution was poured and spread onto a flat glass petri dish, then the films were immersed in water overnight in a low-humidity chamber at room temperature. The dried glass substrates were dipped vertically into the ((ClPPTA)_m_-R′) solutions and withdrawn slowly at the rate of 50 mm/min under ambient conditions, then the coated substrates were dried under vacuum.

### 2.4. Sizing of Fibers Preparation

The sizing agents were prepared with different solid content (1.0–2.0%) of ((ClPPTA)_m_-R′) dissolved in NMP/LiCl. The aramid fibers were immersed in the sizing agent for 10 min, then washed with deionized water and dried in vacuum at 60 °C for overnight, affording sizing fibers. 

### 2.5. Characterization

Fourier transform Infrared spectra (FT-IR) of the ((ClPPTA)_m_-R′)were measured by using an FT-IR spectrometer (Nicolet 8700, Thermo Scientific, Waltham, MA, USA). Solid-state ^13^C CP/MAS nuclear magnetic resonance spectroscopy was recorded on a Bruker spectrometer (Bruker Avance 400, Bruker, Zurich, Switzerland) operating at 100.61 MHZ under MAS conditions. X-ray diffraction (XRD) patterns of the ((ClPPTA)_m_-R′) were conducted on an X-ray diffractometer (D/Max-2550 PC, Rigaku Co., Akishima, Japan) to measure their crystalline-related properties. Thermal gravimetric analysis (TGA, TG209F1 Iris, Netzsch, Exton, PA, USA) was carried out for the measurement of thermal degradation. The sample was heated in the N_2_ atmosphere at 10 °C/min from room temperature to 800 °C, and then cooling down to room temperature. The wettability of the hydroxyl and ethylene-functional aromatic polyamides films was evaluated by measuring the contact angle of the water according to the sessile drop technique implemented on a Dataphysics OCA 40 contact angle system. For contact angle measurements, the aramid film was fixed on a frame, the volume of the water droplets was 5 μL, the contact angle value showed was an average of five measurements. The surface chemical composition of the aramid fibers was estimated by XPS on a RBD upgrade PHI-5000C ESCA system (Fremont, CA, USA). The XPS spectra was provided Mg Kα radiation (*h* = 1253.6 ev) power 250 w, high voltage 14.0 kv with a detection angle at 54°. The base pressure of the analyzer chamber was about 5 × 10^−^^8^ Pa. The sample was directly pressed to a self-supported disk (10 × 10 mm^2^) and mounted on a sample holder, then transferred into the analyzer chamber. The whole spectra (0–1000 ev [1200] ev) and the narrow spectra of all the elements with much high resolution were both recorded using RBD 147 interface (RBD Enterprises).A scanning electron microscope (FE-SEM, Su8010, Hitachi, Japan) was employed to analyze the surface morphology of unsized and sized aramid fibers. The interfacial shear strength of the fiber/epoxy composites was evaluated by the micro-bond technique, the vinyl epoxy resin mixture was dropped on the aramid fibers, and the vinyl epoxy droplets were formed naturally on the single filament, then the specimens were placed into a vacuum oven and kept at 80 °C for 12 h. The micro-bond tests were performed on an XQ-1 fiber tensile testing machine with an upper clamp displacement rate of 0.1 mm/min. The value of the interfacial shear strength (IFSS) was carried using the following equation:

IFSS = *F*/ΠdL (1)

where *F* is the maximum pullout force, d is the fiber diameter, and L is the embedded length of the aramid fiber in vinyl epoxy resin. The average value of the samples showed was an average of 30 tests. 

## 3. Results and Discussion

### 3.1. Molecular Structure

**Figure 1:**
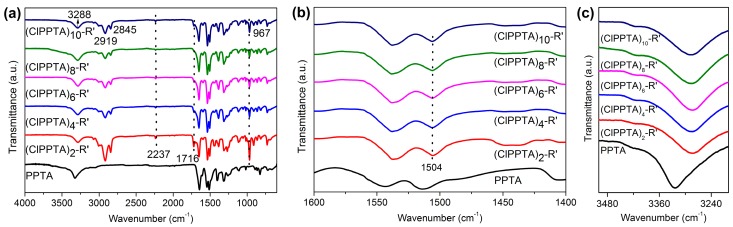
FT-IR spectra of the Poly(*p*-phenylene terphthalamide) (PPTA) and ((ClPPTA)_m_-R′) in the wavenumber range of (**a**) 600–4000 cm^−1^, (**b**) 1400–1600 cm^−1^ and (**c**) 3500–3200 cm^−1^.

FT-IR and solid-state ^13^C NMR spectroscopic techniques were used to investigate the chemical structures of the hydroxyl and ethylene groups in functional ((ClPPTA)_m_-R′) polyamides, respectively. The FT-IR spectra of the solid powders of PPTA and ((ClPPTA)_m_-R′) are shown in Figure 1. Pure PPTA exhibited a broad absorption peak at 3324 cm^−1^ attributed to hydrogen-bonded N–H groups, 1648, 1512, and 1312 cm^−1^ corresponding to amide I, II, and III deformation vibrational bands, respectively. Although, the overall FT-IR spectra of the ((ClPPTA)_m_-R′) were quite similar to that of the pure PPTA, the ((ClPPTA)_m_-R′) exhibited new absorptions at 2917, 2844, 2237, 1716, and 967 cm^−1^, indicating the presence of CH_2_, CN, O–C=O, and CH=CH of the ((ClPPTA)_m_-R′) (Figure 1a). Moreover, localized enlargement of FT-IR spectrum exhibited a new absorption at 1504 cm^−1^ (Figure 1b), revealing the presence of Cl groups. These results indicated that the successful synthesis of the hydroxyl and ethylene-functional aromatic polyamides. It is known that the N–H stretching vibration area of polyamides spans a range from 3100 to 3500 cm^−1^. As shown in Figure 1c, the PPTA and the ((ClPPTA)_m_-R′) exhibited two infrared bands at 3421/3324 and 3413/3288 cm^−1^, which are deemed to “free” and hydrogen-bonded N–H stretching modes, respectively [33], which also indicated the ((ClPPTA)_m_-R′) existed intermolecular hydrogen bonds.

**Figure 2:**
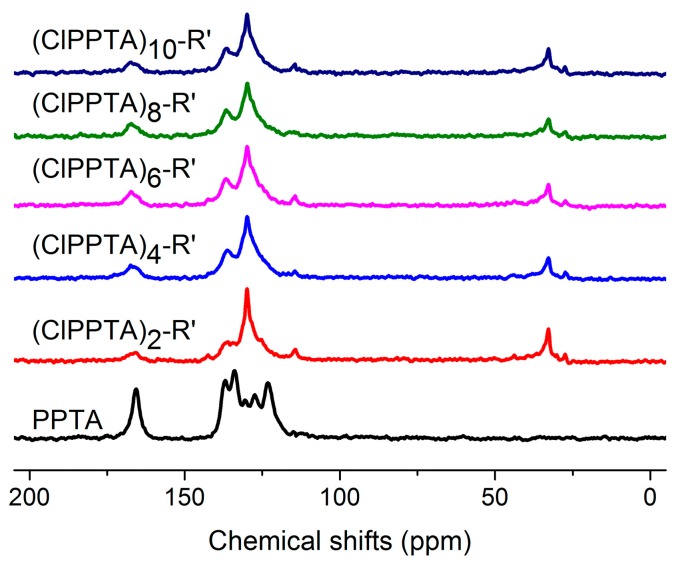
Solid state CP/MAS ^13^C NMR spectra of the PPTA and the hydroxyl and ethylene-functional aromatic polyamides.

Moreover, the ^13^C NMR spectra are further adopted in determining the chemical structure of hydroxyl and ethylene groups in functional aromatic polyamides. As shown in Figure 2, the solid state CP/MAS ^13^C spectra of the PPTA and ((ClPPTA)_m_-R′) are similar to a certain extent. For the ((ClPPTA)_m_-R′), the peak at 32.8 ppm is attributed to the carbon atoms of CH_2_/CH from the hydroxyl-terminated liquid nitrile rubber, the carbon of C–N bonds of the hydroxyl-terminated liquid nitrile rubber is demonstrated at 114.2 ppm. In addition, the ((ClPPTA)_m_-R′) shows two peaks at 129.7 and 136.7 ppm, which are assigned to the carbon atoms on the phenyl ring and the CH=CH of hydroxyl-terminated liquid nitrile rubber, respectively. The peak exhibited at 167.5 ppm is assigned to C=O. All this information confirms that hydroxyl and ethylene-functional aromatic polyamides have been successfully synthesized.

### 3.2. Crystalline Structures

**Figure 3:**
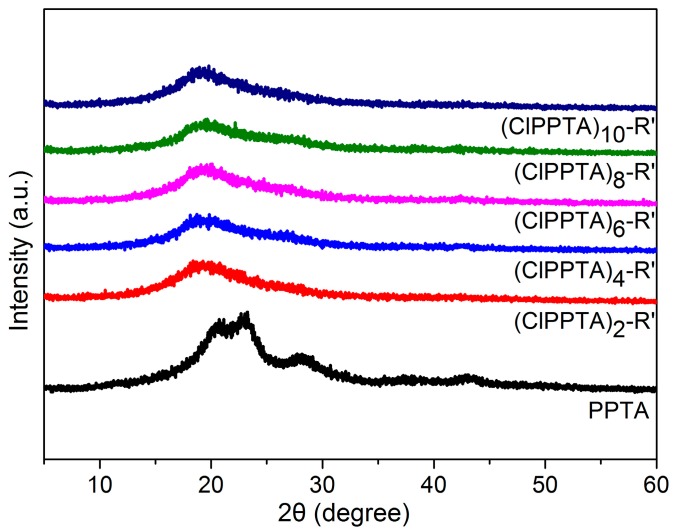
X-ray powder diffraction of the PPTA and hydroxyl and ethylene-functional aromatic polyamides, respectively.

The wide-angle X-ray powder diffraction patterns of the PPTA and ((ClPPTA)_m_-R′) are exhibited in Figure 3. PPTA showed three broad diffraction peaks, observed at 2θ = 20.7° (*d* = 0.42 nm), 23.1° (*d* = 0.38 nm), and 28.4° (*d* = 0. 31 nm), owing to (110), (200), and (004) planes, respectively. In the case of hydroxyl and ethylene-functional aromatic polyamides, only one diffraction peak at 2θ = 19.7° was observed, indicating a completely amorphous packing of macromolecules. It can be explained that the chlorine substituent disrupted the regularity and crystalline of the hydroxyl and ethylene-functional aromatic polyamides. In addition, the introduction of hydroxyl-terminated liquid nitrile rubber leads to a decreased rigidity of the ((ClPPTA)_m_-R′).

### 3.3. Thermal Stability

**Figure 4:**
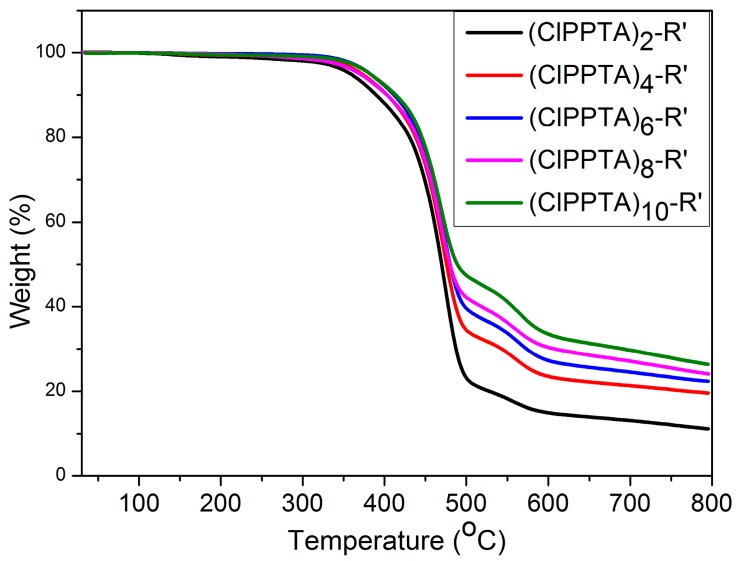
TGA curves of the hydroxyl and ethylene-functional aromatic polyamides, respectively.

**Scheme 2:**
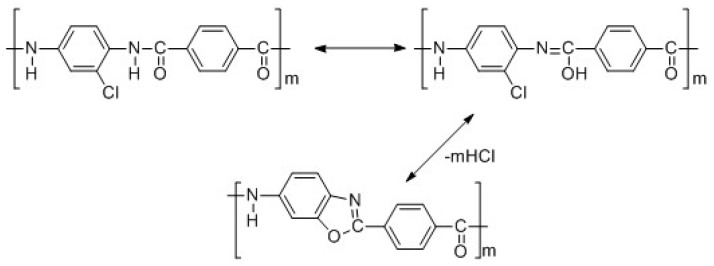
Possible mechanism of ring-forming reaction in hydroxyl and ethylene-functional aromatic polyamides i.e., ((ClPPTA)_m_-R′).

Figure 4 shows the TGA thermograms of the ((ClPPTA)_m_-R′), which were carried out at 10 °C/min under a nitrogen gas environment. The hydroxyl and ethylene-functional aromatic polyamides exhibited two-step decomposition as the temperature increases. The first stage (320–500 °C) weight loss of the ((ClPPTA)_m_-R′) resulted from the chorine groups, which can decrease the regularity and lower the intermolecular order. Additionally, flexible rubber chains can also lower thermal stability. Kapuscinski et al [34] proposed the degradation mechanism of the halogenated polymers, as shown in Scheme 2. The first stage of the weight loss is related to the degradation of HX during the reaction between the halogen and the neighboring amide bonds, as well as the leakage of rubber. The second stage (500–800 °C) of the weight loss is attributed to the degradation of the heterocyclic polymers. Simultaneously, we found that the stability of the ((ClPPTA)_m_-R′) increases with the increasing amount of the molecular chain segments. In addition, the char yield of the ((ClPPTA)_m_-R′) also increases with the increasing amount of the molecular chain segments. The reason might be that the higher fraction of (ClPPTA)_m_ would increase the molecular weight of ((ClPPTA)_m_-R′). 

### 3.4. Solubility

Table 1 listed the solubility of the PPTA and ((ClPPTA)_m_-R′) in different solvents. Note that the ((ClPPTA)_m_-R′) is insoluble in concentrated H_2_SO_4_ because the rubber segments could be carbonized in concentrated sulfuric acid. However, the ((ClPPTA)_m_-R′) could be partially soluble in polar aprotic organic solvents such as NMP, DMAc, DMF and DMSO at 80 °C, and even became completely soluble when a small amount of LiCl was added. This is in big contrast to PPTA since the PPTA is only soluble in sulfuric acid, indicating much better solubility benefited from the functionalization. It is possible that a chlorine group is introduced on the phenyl ring of PPTA destroying the intermolecular hydrogen bonding and rigid backbone. We believe the improvement of solubility should be much helpful to enhance the processability of the aromatic polyamides. 

### 3.5. Wettability

**Figure 5:**
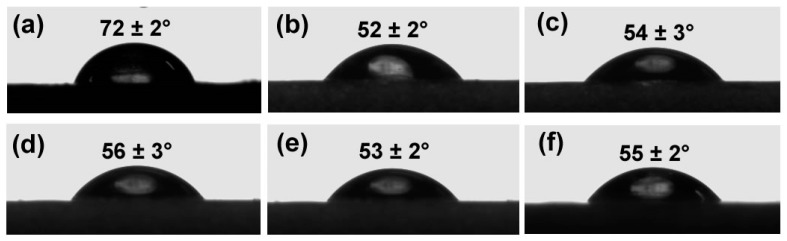
PPTA films (**a**) and static contact angle tests of the hydroxyl and ethylene-functional aromatic polyamides i.e., ((ClPPTA)_m_-R′) films (**b**–**f**).

From the point of industrialization, the good wetting behavior of the film surface plays a significant role in improving the miscibility of the interface. Figure 5 shows the static water contact angle tests of the PPTA and ((ClPPTA)_m_-R′) films, respectively. The contact angles of the ((ClPPTA)_m_-R′) films (Figure 5b–f) are much lower than that of PPTA films (Figure 5a), implying that the hydroxyl and ethylene functional groups would highly improve the wettability and affinity of aromatic polyamides toward the water. Simultaneously, it can be found that the contact angles of ((ClPPTA)_m_-R′) films (Figure 5b–f) didn’t vary significantly. It is probably due to the size of the contact angle being related to the introduction of the active functional group and has nothing to do with the molecular weight. 

### 3.6. Surface Chemical Composition of the Aramid Fibers

**Figure 6:**
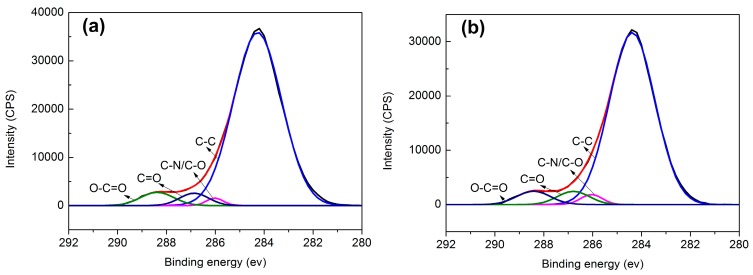
C 1s spectra of unsized (**a**) and sized (**b**) aramid fibers.

XPS measurements can be powerful approaches to analyze the surface compositions and the introduction of additional functional groups in aramid fibers quantitatively [35]. Table 2 lists the surface compositions of aramid fibers sized using 1.5 wt % ((ClPPTA)_2_-R′) LiCl/NMP solution. It can be found that the contents of C atoms in the sized fibers were decreased, while the contents of O and N atoms increased, indicating the good bonding of the resin. The atomic ratio of O/C increased from 0.26 to 0.29, indicating that the sizing agent was successfully introduced onto the surface of aramid fibers. Figure 6a,b shows C1s level XPS spectra of aramid fibers before and after sized by ((ClPPTA)_2_-R′). The unsized aramid fibers showed four peaks with binding energy at 284.4, 286.1, 286.8, and 288.4 eV owing to C–C, C–N, C–O, and O–C=O species, respectively. Upon sized aramid fibers, the contents of polar groups such as C–N/C–O and C=O obviously increased (Table 3), further confirming that the sizing agents were successfully introduced into the surface of aramid fibers.

### 3.7. Surface Morphologies of Aramid Fibers

**Figure 7:**
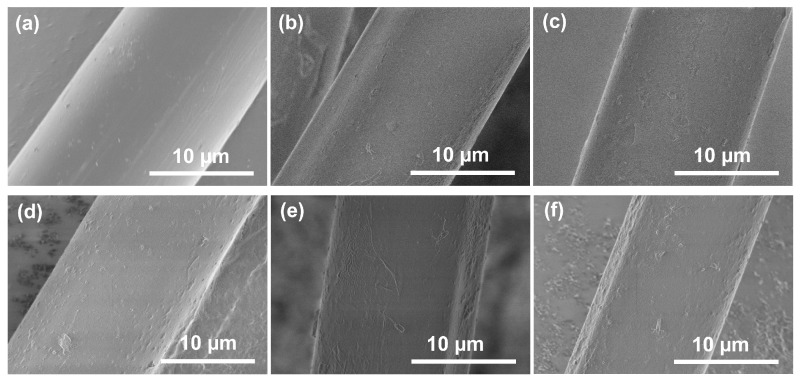
SEM images of (**a**) unsized aramid fiber (AF) and (**b**–**f**) the sized ones: (**b**) AF-(ClPPTA)_2_-R′-1.5%; (**c**) AF-(ClPPTA)_4_-R′-1.5%; (**d**) AF-(ClPPTA)_6_-R′-1.5%; (**e**) AF-(ClPPTA)_8_-R′-1.5%; and (**f**) AF-(ClPPTA)_10_-R′-1.5%.

The surface morphologies of aramid fibers upon sizing with hydroxyl and ethylene-functional polyamides i.e., ((ClPPTA)_m_-R′) were investigated by SEM. The unsized aramid fibers (Figure 7a) displayed a relatively clean and smooth surface, indicating that a low interfacial adhesion existed between the fibers and matrix. While the aramid fibers sized with 1.5 wt % ((ClPPTA)_m_-R′) LiCl/NMP solution (Figure 7b–f) displayed a rougher surface and some spots were even observed on their surfaces. The results were in coincidence with that of XPS, indicating the uniform sizing layer was formed on the surface of aramid fibers. It is believed that the surface roughness of aramid fiber is beneficial to improve the interfacial properties of composites.

### 3.8. Interfacial Adhesion of Vinyl Epoxy Resin to Aramid Fibers

**Figure 8:**
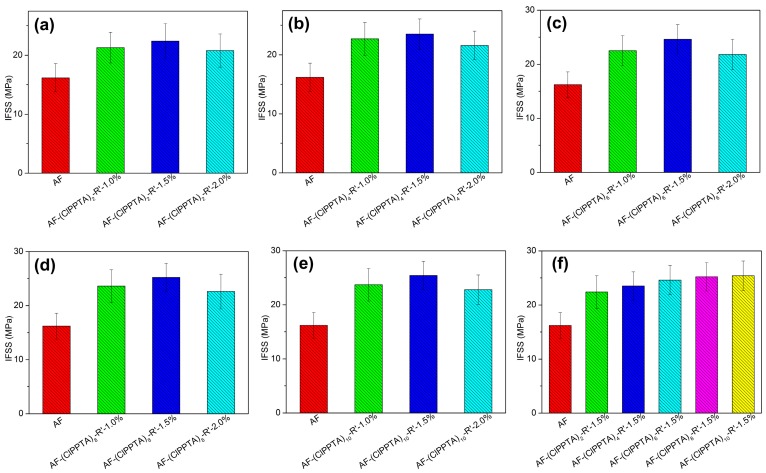
Influence of molecular weight and concentration of ((ClPPTA)_m_-R′) on the IFSS properties of aramid fibers and vinyl epoxy composites: (**a**–**e**) in different solid content of ((ClPPTA)_m_-R′) (1.0–2.0%) dissolved in NMP/LiCl as the sizing agents of aramid fibers to impact on the IFSS of vinyl epoxy composites; (**f**) the same of solid content of ((ClPPTA)_m_-R′) (1.5%) dissolved in NMP/LiCl as the sizing agents of aramid fibers to impact on the IFSS of vinyl epoxy composites

The micro-debond tests were used to investigate the influence of ((ClPPTA)_m_-R′) on the interfacial adhesion properties of the aramid fibers and vinyl epoxy composites. The interfacial shear strength (IFSS) of the composites reinforced with fibers and sized with various weight concentrations was compared. With adjusting the molecular weight and concentrations of the sizing agent i.e., ((ClPPTA)_m_-R′), all the composites showed the highest IFSS values when 1.5 wt % concentration of ((ClPPTA)_m_-R′) was applied (Figure 8a–e). Meanwhile, it appears the higher molecular weight of ((ClPPTA)_m_-R′) led to higher IFSS values at an optimal concentration of the sizing agent (1.5 wt %), as shown in Figure 8f. The reason may be that the higher affinities toward both fibers and vinyl epoxy resin and also its relatively high molecular weight contribute the better mechanical properties [22]. Overall, the addition of ((ClPPTA)_m_-R′) as sizing agents could greatly enhance the adhesion properties of the aramid fibers and the vinyl epoxy matrix.

**Figure 9:**
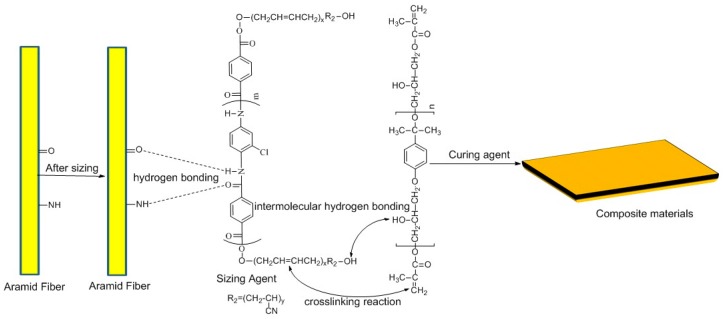
The proposed possible mechanism of the reaction between sized of aramid fibers and vinyl epoxy resin.

To figure out the function of the ((ClPPTA)_m_-R′) sizing agent, we also proposed a possible mechanism, as shown in Figure 9. Upon sizing, the interactions between aramid fibers and sizing agents became more intensive, since intermolecular hydrogen bonding interaction occurred between amide groups of aramid fibers and hydroxyl functional groups of ((ClPPTA)_m_-R′). Meanwhile, CH=CH group in the ((ClPPTA)_m_-R′) would undergo a cross-linking reaction with vinyl epoxy resin in the presence of curing agents. In addition, the intermolecular hydrogen bond could happen between hydroxyl groups of ((ClPPTA)_m_-R′) and hydroxyl groups of epoxy resin, resulting in a more homogenous composites. 

## 4. Conclusions

A series of hydroxyl and ethylene-functional aromatic polyamides ((ClPPTA)_m_-R′) has been synthesized by a low-temperature polycondensation approach. The chemical structure of the ((ClPPTA)_m_-R′) was confirmed by FT-IR and solid-state ^13^C CP/MAS NMR spectra. The ((ClPPTA)_m_-R′) are completely amorphous and exhibit excellent solubility in organic solvents such as NMP, DMAc, DMF, and DMSO with the presence of LiCl. Contact angle measurements indicated with the introduction of hydroxyl and ethylene-functional groups in aromatic polyamides could largely improve the wettability and affinity with water. As-prepared aramid fibers/vinyl epoxy composites exhibited the highest interfacial shear strength when 1.5 wt % of ((ClPPTA)_m_-R′) was added. We believe the synthesis of hydroxyl and ethylene-functional aromatic polyamides opens a new avenue to improve the mechanical properties of aramid composites.

## Figures and Tables

**Table 1:** The solubility of PPTA and ((ClPPTA)_m_-R′) in different solvents.

Sample	PPTA	((ClPPTA)_m_-R′)
H_2_SO_4_	++	−
NMP	−	+−
DMAc	−	+−
DMSO	−	+−
DMF	−	+−
THF	−	−
CHCl_3_	−	−
NMP + 0.5% LiCl	−	++
DMAc + 0.5% LiCl	−	++
DMF + 0.5% LiCl	−	++
DMSO + 0.5% LiCl	−	++

Note: ++: Highly soluble at 80 °C; +: soluble at 80 °C; +−: partially soluble at 80 °C; −: insoluble.

**Table 2:** Surface element analysis of aramid fibers.

Samples	Chemical composition (at %)			Atomic ratio	
	C	N	O	O/C	N/C
Unsized fibers	78.4	1.2	20.4	0.26	0.015
Sized fibers	76.6	1.4	22.0	0.29	0.018

**Table 3:** Deconvolution analyses of C 1s Peaks of unsized and sized aramid fibers.

Sample	Content of functional groups (%)			
	C–C	C–N/C–O	C=O	O–C=O
Unsized fibers	89.8	1.4	3.9	4.8
Sized fibers	87.8	2.5	4.7	5.1
